# Hypothyroidism and Depression: A Narrative Review

**DOI:** 10.7759/cureus.28201

**Published:** 2022-08-20

**Authors:** Surya P Nuguru, Sriker Rachakonda, Shravani Sripathi, Mashal I Khan, Naomi Patel, Roja T Meda

**Affiliations:** 1 Internal Medicine, Kamineni Academy of Medical Sciences and Research Center, Hyderabad, IND; 2 Surgery, Bogomolets National Medical University, Kyiv, UKR; 3 Surgery, Bhaskar Medical College, Hyderabad, IND; 4 Internal Medicine, Khyber Girls Medical College, Peshawar, PAK; 5 Research, Smt. Nathiba Hargovandas Lakhmichand (NHL) Municipal Medical College, Ahmedabad, IND; 6 Medicine, Narayana Medical College, Nellore, IND

**Keywords:** treatment-resistant depression, thyroid replacement therapy, thyroid peroxidase antibodies, subclinical hypothryroidism, depression in hypothyroidism, levothyroxine, depression, hypothyroidism

## Abstract

There has been an established relationship between hypothyroidism and depression. Studies have demonstrated that somatostatin and serotonin influence the hypothalamus-pituitary-thyroid axis, which links hypothyroidism to depression. Multiple studies concluded that undiagnosed, untreated, undertreated patients with hypothyroidism are at increased risk of developing depression. Autoimmune thyroiditis is also associated with an increased risk of depression. Elevated thyroid-stimulating hormone (TSH), antithyroglobulin (TgAb), and thyroid peroxidase antibodies (TPOAb) levels have all been linked to depression and an increased risk of suicide. Moreover, hypothyroidism is known to be one of the leading causes of treatment-resistant depression. Treating underlying hypothyroidism with thyroid replacement therapy could significantly improve mood disorders such as depression. Levothyroxine therapy is also used as adjunctive therapy to antidepressants in the management of depression, and it is known to improve the symptoms of depression rapidly when compared to antidepressants alone. This review strengthens the link between hypothyroidism and depression, and it also demonstrates how treating the underlying hypothyroidism in people who have been diagnosed with depression will be very beneficial.

## Introduction and background

Depression is a serious illness that has a life-time risk of occurrence of 20% in the United States [1]. Many factors increase a person's risk of developing depression, and these include both modifiable and non-modifiable factors such as genetics, hormonal disturbances, and association with other medical disorders [2]. One such common association exists between hypothyroidism and depression. The relationship between hypothyroidism and depression was first described in 1825 by Parry, who noted increased "nerve strokes" in thyroid disease. Seagull discovered a link between myxoedema and psychosis in 1873, which was later established by the committee of the clinical society in 1888. The term "myxedema madness" was introduced by Asher in 1949 to represent the mental state changes in hypothyroid patients [3]. Both an increase or decrease in the thyroid hormones can result in mood disorders like depression and anxiety, which can easily be resolved by addressing the thyroid imbalance. Overt hypothyroidism is seen in 1-4% of people with affective disorders, whereas subclinical hypothyroidism (SCH) is found in 4-40% [4]. Cleare et al. in 1995 conducted a study on 20 patients which showed that 40% of patients with hypothyroidism develop clinically significant depression [5]. According to the latest research, metabolic abnormalities in the brain, which result in disordered neurotransmission, behavior, and cognition, play a key role in the etiology of depression [6,7]. Depression inhibits the hypothalamo-pituitary-thyroid (HPT) axis. The HPT axis interacts with aminergic systems, which have been linked to depression [8]. Clinically effective adjunctive therapy with levothyroxine (L-T4) or T3 has been shown to restore alterations in glucose metabolism in depression. It is probable that one of the reasons for depression is a weakened metabolic action of thyroid hormones in the brain [6,7]. In this review, we aim to elaborate on the current understanding about the relationship between hypothyroidism and depression.

## Review

Hypothalamic-pituitary-thyroid axis

**Figure 1:**
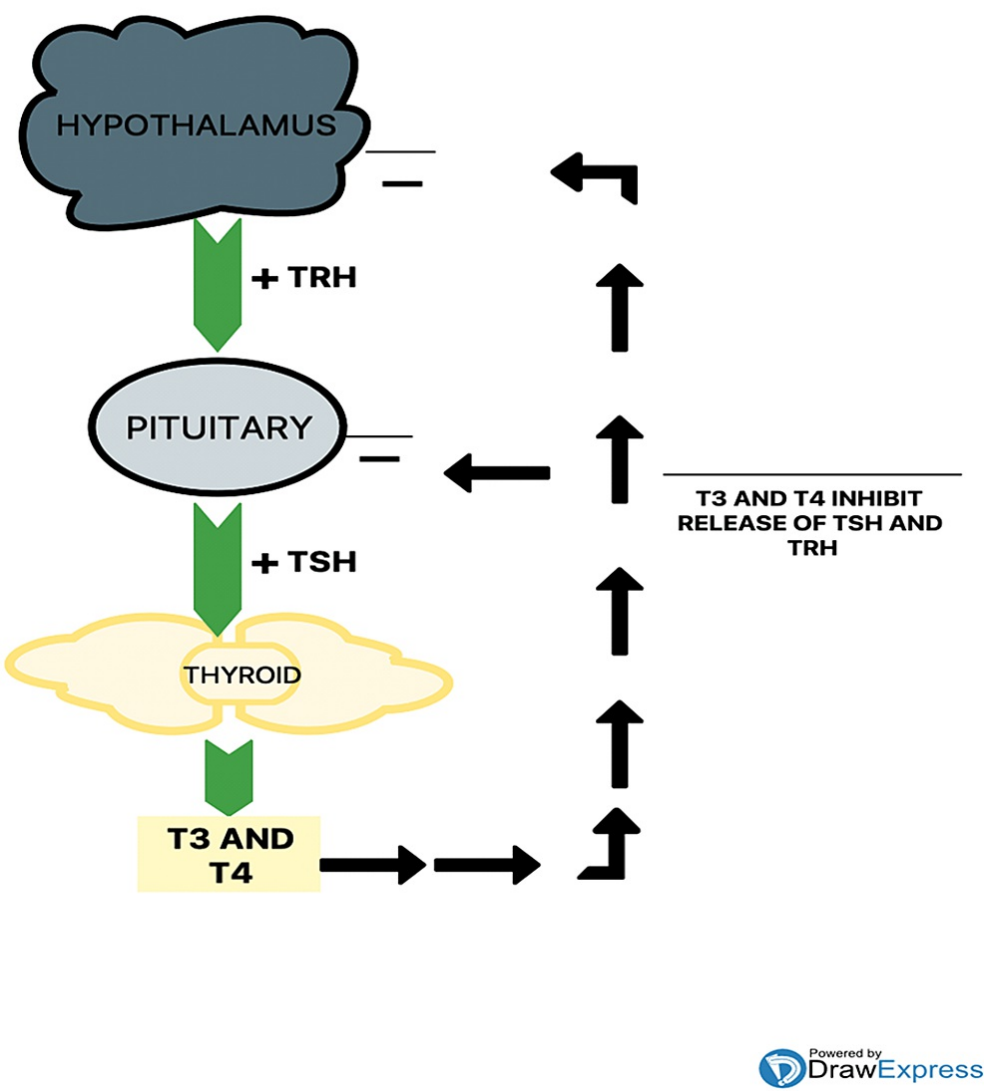
Hypothalamic-pituitary-thyroid axis T3: tri-iodothyronine, T4: thyroxine, TRH: thyrotropin releasing hormone, TSH: thyroid stimulating hormone.

About 0.2-5.3% of Europeans and 0.3-3.7% of Americans suffer from hypothyroidism [9]. The American Association of Endocrinologists states that depression must be considered in all patients diagnosed with subclinical or clinical hypothyroidism [10]. Moreover, the most common neuropsychiatric manifestations of thyroid disorders are depression and cognitive impairment [11]. The HPT axis (Figure 1) is full of complicated interactions between components such as the thyroid hormone, deiodinase enzymes, transporter proteins, and receptors [12]. Thyroid hormone secretion is regulated by the thyroid-stimulating hormone (TSH) from the pituitary, which is stimulated by thyrotropin-releasing hormone (TRH). Thyroid hormones downregulate thyroid hormone secretion through a negative feedback mechanism. The thyroid gland secretes 20% of triiodothyronine (T3) directly, while the remaining 80% is derived from the deiodination of thyroxine (T4) [13,14].

Influence of somatostatin and serotonin on HPT axis and depression

Changes in hormone levels such as somatostatin and serotonin in the central nervous system can result in neuropsychiatric disturbances [15]. Existing research also implies that these pathways may influence the HPT axis, explaining the link between subclinical hypothyroidism and depression. Somatostatin levels in the cerebrospinal fluid are lower in those with depression, resulting in higher TSH levels [15,16]. On the other hand, serotonin insufficiency, which is frequent in those who suffer from depression, has been related to changes in the HPT axis such as suppression of TSH release [17].

Autoimmune thyroid disorder and depression

Depression is associated with neuroendocrine disturbances such as thyroid hormone disorders. Missing a diagnosis of subclinical hypothyroidism (SCH) can possibly be a cause of depression or mood cycling, as well as a delayed response to therapy [18]. Because MDD is frequently associated with autoimmune thyroiditis, it might be considered an autoimmune condition or an immune system disorder. Antithyroid antibodies are elevated in many people with depression [12]. Microsomal antibodies are also commonly seen in patients with chronic lymphocytic thyroiditis. Additional thyrotropin receptor (TSHR) antibodies that block TSH function can cause hypothyroidism [8]. A reduced TSH response to TRH has been linked to increased suicide risk, suicidal intent, violent suicide attempts, and greater lethality, mainly in depressed women [19]. TSH, antithyroglobulin (TgAb), and TPOAb levels have all been proposed as potential indicators of suicidality in MDD [19]. Thyroid-binding inhibitory immunoglobulins block TSH from binding to its receptor, causing hypothyroidism to develop. Atypical depression is associated with high levels of immunoglobulins and microsomal antibodies linked to treatment resistance [20].

Furthermore, higher TRH concentrations have consistently been reported in the cerebrospinal fluid of individuals with depression, indicating that depression is associated with a changed TRH response [12,21]. On the other hand, depression has been linked to developing hypothyroidism. The most widely accepted explanation is a thyroid axis disruption, which blunts the TSH response to TRH stimulation [22]. Patients with atypical depression have higher levels of microsomal antibodies and TSH-blocking immunoglobulin while their free triiodothyronine (FT3), free thyroxine (FT4), and TSH levels are within normal limits [21]. Overt hypothyroidism and SCH are common concomitant illnesses associated with MDD, particularly in women [23]. Treatment-resistant MDD, increased severity of MDD, psychotic phenomenology, and somatic symptoms are all possible consequences of this comorbidity [24]. Patients with MDD may have immune system activation, which can develop into autoimmune thyroiditis and thyroid dysfunction [25]. Thyroid hormone replacement therapy has been shown to benefit individuals with hypothyroidism and MDD, particularly treatment-resistant patients or/and those with unusual symptoms [24]. Overt hypothyroidism, on the other hand, is associated with effective and broader neuropsychiatric symptomatology [26]. It has been proposed that subclinical autoimmune thyroid dysfunction may play a causative role in MDD [21].

"Brain hypothyroidism"

According to a reversal hypothesis, the pathogenetic mechanism of MDD might be related to a condition of local cerebral hypothyroidism with normal peripheral thyroid hormone concentrations. Many researchers have used the phrase "brain hypothyroidism" to describe this idea, based on the discovery of type II deiodinase inhibition in the brain and reduced T4 transport across the blood-brain barrier in depressed individuals. Furthermore, functional changes such as loss of nocturnal thyrotropin (TSH) increase, reduced TSH response to thyrotropin-releasing hormone (TRH) stimulation, and mild elevation of serum thyroxine (T4) have been documented [27]. The DeltaDeltaTSH (TSH) test is a better way to detect dysregulation of the HPT axis in MDD [28]. It calculates the difference in TSH response to TRH tests between 23:00 and 08:00 hours on the same day. Therapy resistance was linked to changes in the HPT axis after two weeks of antidepressant treatment. Clinical remission did occur once the HPT axis activity was restored chronobiologically [29]. In individuals with MDD, however, monitoring thyroid axis hormones at different times of the day is not accurate [29].

Hypothyroidism and its association with depression

Lang et al. conducted a cross-sectional study in 2019 on 1706 Chinese patients diagnosed with major depressive disorder (MDD) to investigate the prevalence of severe SCH in patients with MDD. According to the study, those with severe SCH are more likely to attempt suicide and experience psychiatric symptoms. Alongside, it was also seen that severe anxiety, depressive symptoms, and psychotic symptoms, as well as older age and higher BMI, are all possibly related to elevated TSH levels [30]. Ittermann et al. in 2015 conducted a study on 2142 individuals who followed the Study of Health in Pomerania (SHIP-1) and the Life Events and Gene-Environment Interaction in Depression study (LEGEND) to investigate the association between diagnosed thyroid disorders, serum TSH levels, and anti-thyroid-peroxidase antibodies (TPOAbs) with depression and anxiety. The study concluded that untreated diagnosed hypothyroidism was linked to a higher Beck depression inventory (BDI)-II score and anxiety. In contrast, untreated diagnosed hyperthyroidism was linked to a higher risk of MDD in the previous year. Sub-analyses revealed different interactions between childhood maltreatment and thyroid abnormalities, changing the link between depression and anxiety disorders [31]. To assess the prevalence of depression in hypothyroid patients, Mohammad et al. conducted cross-sectional research on patients with hypothyroidism visiting the primary health care and endocrine clinic at King Fahd Hospital of the University (KFHU) in Al Khobar in 2019. According to the study, 33.9% of patients were diagnosed with depression to varying degrees. According to the study's findings, depression is common among hypothyroidism patients. It is suggested that people with hypothyroidism be screened for depression [32]. Demartini et al. surveyed 63 patients with subclinical hypothyroidism (SCH) in the year 2010 in Milan to establish the association of depressive symptoms in patients with SCH. The study demonstrated the prevalence of depression to be 63.5% among patients with SCH and suggested the importance of a psychiatric evaluation in patients diagnosed with SCH [33]. Similarly, a study by Loh et al. on 12,315 patients in 2019 also demonstrated the link between SCH and depression. Thus, it may be said that those who have SCH are more likely to have depression (relative risk 2.35, 95% confidence intervals [CI], 1.84 to 3.02; p < 0.001) than those who do not, and that early and regular screening is essential to lower morbidity and mortality. Furthermore, a subanalysis found that the geriatric group had a greater incidence of depression [34] (Table 1).

**Table 1:** Studies demonstrating the association between depression and hypothyroidism SCH: subclinical hypothyroidism; MDD: major depressive disorder; BMI: basal metabolic index; TSH: thyroid stimulating hormone; TPO-Abs: thyroid peroxidase antibodies.

Author’s name	Type of study	Year	Number of patients	Aim	Conclusion
Lang et al. [30]	Cross sectional study	2019	1,706	To investigate the prevalence of SCH in patients diagnosed with MDD	Severe anxiety, depressive and psychotic symptoms, as well as older age and higher BMI are possibly related to elevated TSH levels.
Ittermann et al. [31]	N/A	2015	2,142	To investigate the association between diagnosed thyroid disorders (TSH, TPO-abs) with depression and anxiety.	This study concluded that untreated diagnosed hypothyroidism was linked to a higher BDI-II score and anxiety, while untreated diagnosed hyperthyroidism was linked to a higher risk of MDD in the previous year.
Mohammad et al. [32]	Cross sectional study	2019	N/A	To estimate the prevalence of depression in hypothyroid patients.	The study concluded that depression is prevalent among patients diagnosed with hypothyroidism and it recommended screening for depression in patients suffering from depression.
Demartini et al. [33]	N/A	2010	63	To estimate the rate of association of depressive symptoms in patients with SCH.	The results of the study suggested a prevalence of depression to be 63.5% among patients with SCH.
Loh et al. [34]	N/A	2019	12,315	To demonstrate the association between SCH and depression.	The study concluded that individuals with SCH are more prone to develop depression than those without, and that early and frequent screening is crucial to reduce morbidity and mortality.

Hypothyroidism and its effect on health related quality of life

In the year 2021, Diaz et al. did research to determine the association between TSH levels and health related quality of life (HRQL). Hypothyroid individuals who had received sufficient treatment for hypothyroidism were included in the research. HRQL was measured using the specific thyroid disease ThyPRO-39 questionnaire in 218 consecutive patients with primary hypothyroidism of any cause who attended an endocrinology department in a single center. The study's findings revealed that increased TSH levels resulted in a worsening of HRQL. TSH has also been found to be linked to ratings of tiredness and emotional sensitivity assessments [35]. Shivaprasad et al. in 2018 did research to measure HRQL in hypothyroid individuals. The SF-36 questionnaire was used to examine 244 individuals in the research, and all of the individuals in the trial were older than 18 years. Six of the eight SF-36 measures had significantly lower scores for hypothyroidism patients compared to healthy controls in the research. There were no significant intergroup variations in the domains of "role effective" and "social functioning" [36]. In 2016, Winther et al. published research that looked at disease-specific (ThyPRO) and general (SF-36) HRQL in those who had hypothyroidism owing to autoimmune thyroiditis after initiating levothyroxine therapy. A prospective cohort study was conducted on 78 individuals at two Danish university hospitals' endocrine clinics. According to ThyPRO and SF-36, HRQL was considerably impaired in this group of hypothyroid patients before treatment, with fatigue being the most significant impairment. Several aspects of HRQL improved throughout the first six months of LT4 therapy, albeit complete recovery was not achieved [37]. Tan et al. researched Asian patients in 2019 to see if there was a relationship between hypothyroid symptoms, comorbidities, quality of life (QOL), and hypothyroid-related symptoms in patients on LT4 medication. In Asian hypothyroid individuals, weight gain, weariness, weakness, dry or coarse skin, leg edema, and increased comorbidities and symptoms were all linked to a decreased QOL [38].

Treatment of hypothyroidism

The preferred treatment for hypothyroidism is levothyroxine monotherapy in solid form, given on an empty stomach. Treatment should begin when clinical symptoms of hypothyroidism are present, as well as laboratory proof of overt hypothyroidism. There is no reason to avoid prescribing generic preparations, and switching between levothyroxine brands in stable individuals is not advised [39]. In overt hypothyroidism, the optimum daily dosage is 1.5-1.8 μg per kg of body weight [39-41]. The beginning dose for individuals with coronary artery disease is usually 12.5-25.0 μg per day, and it should be progressively raised based on symptoms and TSH levels. TSH levels are measured after 4-12 weeks of medication, every six months, and finally annually if the patient is stable. Adjustments should be made based on laboratory findings, considering that modest dosage adjustments might significantly impact serum TSH concentrations in some individuals (e.g., those with low body weight or those who are elderly). Despite normal TSH values, low triiodothyronine concentrations in certain people have little therapeutic importance. Routine tri-iodothyronine measurements should not be used to evaluate therapy success [42].

Role of levothyroxine therapy in patients with hypothyroidism and depression

In 2020, Moon et al. conducted a single-blinded study on 23 individuals with hypothyroidism who were 65 years or older and on steady dosages of levothyroxine replacement medication. According to the findings, increasing the LT4 dosage in older people getting thyroid hormone replacement improves depressive mood without inducing significant hyperthyroid symptoms or signs. These findings suggest that the hypothyroidism treatment goal should be changed based on the mental status and that low-dose LT4 medicine might be used in conjunction with depression therapy [43]. In 2017, Krysiak et al. performed a study on males with hypothyroidism. Men with overt autoimmune hypothyroidism were placed in group A, men with subclinical autoimmune hypothyroidism were placed in group B, and healthy euthyroid men without autoimmune hypothyroidism were placed in group C. The researchers wanted to learn more about sexual function and depression in males with autoimmune hypothyroidism. The study found that males with autoimmune hypothyroidism had sexual and mood issues and that L-thyroxine medication helps hypothyroid people with sexual dysfunction and depressive symptoms [44]. In 2014, Vishnoi et al. researched individuals with SCH to see if levothyroxine might help patients with depression and SCH. The study group consisted of 300 SCH patients, whereas the control group consisted of 300 healthy patients with sex, age, and gender-matched research group. The study concluded that taking levothyroxine improves the Hamilton Depression Rating Scale (HAM-D) significantly, emphasizing the importance of thyroid screening in patients with low mood and implying the use of levothyroxine therapy in patients with SCH and depression [45]. Harten et al. reviewed research in 2008 to study if depressive symptoms in SCH patients should be treated with thyroid hormone replacement therapy. The study includes three different randomized control trials. None of these investigations focused on depressed individuals but on patients with SCH, with the depression score scale as a secondary outcome. The researchers found insufficient evidence to support the use of levothyroxine treatment in individuals with depression and SCH [46] (Table 2).

**Table 2:** Studies showing role of levothyroxine therapy in the management of depression in hypothyroidism LT4: levothyroxine; HAM-D: Hamilton Depression Rating Scale; SCH: subclinical hypothyroidism.

Author	Year	Aim	Conclusion
Moon et al. [43]	2020	To show reversal of depressive symptoms with levothyroxine therapy in patients with hypothyroidism.	Low-dose LT4 medication could be used as a supplement to depression treatment
Krysiak et al. [44]	2017	To investigate sexual functioning and depression in men with autoimmune hypothyroidism.	Sexual and mood disorders are present in men with autoimmune hypothyroidism and L-thyroxine treatment benefits hypothyroid individuals with sexual dysfunction and depressive symptoms.
Vishnoi et al. [45]	2014	To demonstrate benefits of levothyroxine therapy on depressive symptoms in hypothyroid patients.	Administration of levothyroxine is associated with an significant improvement on HAM-D.
Harten et al. [46]	2008	Efficacy of hormone replacement therapy in management of depression in hypothyroid patients,	There was lack of evidence that proves beneficial effect of levothyroxine therapy in patients with depression and SCH.

It has been postulated that T3 and T4 mediate serotoninergic effects, most probably through desensitization of the 5-HT1A autoreceptor [47]. Multiple open-labeled studies have been performed, showing an improvement in depression when thyroid hormones have been used as an adjunctive therapy. In particular, thyroxine (T4) is used with lithium in rapid-cycling bipolar disorder, a condition in which depression and mania rapidly alternate. In depression, L-triiodothyronine (T3) has been used in preference to T4 because of its rapid onset and offset of action. In women starting treatment, T3 hastens the onset of therapeutic action of standard antidepressant drugs, whereas this effect is not seen in men [48]. Although results are inconsistent, triiodothyronine (T3), administered from 20 to 65 μg/day, can augment the effects of tricyclic antidepressants and sertraline for treating major depression [49,50]. Treatment with a supraphysiologic dose of LT4 (250-600 μg/day) has also been effective in patients with bipolar disorder who are unresponsive to standard therapies [27,51,52], especially during the refractory depression phase [53]. Those previous studies used thyroid hormone as an adjunct to conventional antidepressant agents with a much higher thyroid hormone dose than that required to treat primary hypothyroidism. Moreover, in elderly patients, a high dose of thyroid hormone can result in severe adverse effects, such as cardiovascular and skeletal side effects. However, there has been an improvement in depression significantly in older patients with hypothyroidism with only a minimal increase in LT4 dose (12.5 μg/day) without antidepressant agents [43]. Therefore, it is evident that thyroid hormone replacement therapy improves depression in hypothyroid patients while also can themselves be used adjunctively in patients with MDD and no thyroid disorders.

Limitations of the study

This article is a comprehensive review of the literature on the connection between hypothyroidism and depression and is based on publications found on PubMed and Google Scholar. Since hypothyroidism and depression are vast topics, we could focus only on limited parts of them. All pertinent data could not be assessed because this topic has been the subject of numerous trials and meta-analyses. Our objective was to study the association between depression and hypothyroidism and to strengthen the association. While there are various studies that establish an association between them, there are also studies that show no relation. Our study was limited to the studies done in the past, and a few of the studies were not conclusive. This topic requires further research to establish a solid causative relationship.

## Conclusions

As stated above, a vast body of research supports the association between hypothyroidism and depression. Patients with hypothyroidism commonly experience depression as a comorbidity, with the majority of cases being treatment-resistant depression. Several causes, including the effect of thyroid hormones on the HPT axis, have contributed to this. On the other hand, depressed patients have been found to have an underlying hypothyroid status. Hence, screening for hypothyroidism in patients suffering from depression is necessary. The treatment protocol usually entails the treatment of hypothyroidism appropriately with thyroid replacement therapy and antidepressants. Levothyroxine functions to improve the action of antidepressants, potentiating their effects in treating and improving the symptoms of depression. Moreover, it has also been established that T3 and T4 can be used as adjunctive therapies to manage major depressive disorders in euthyroid patients. Hence, confirming the role of thyroid hormones in maintaining mood stability in the presence or absence of SCH. Further research is needed to establish proper therapeutic guidelines and strengthen the link between hypothyroidism and depression.
